# Safety and clinical outcomes of a first-in-human trial of point-of-care manufactured trispecific CAR T cells targeting CD19, CD20, and CD22

**DOI:** 10.21203/rs.3.rs-8605291/v1

**Published:** 2026-02-09

**Authors:** Sumithira Vasu, Nathan Denlinger, No-Joon Song, Danielle Elsberry, Qiuhong Zhao, Lianbo Yu, Evandro Bezerra, Nicole Szuminski, Dina Schneider, Pradyot Dash, Louisa Wirthlin, Narendranath Epperla, Yazeed Sawalha, Jennifer Woyach, Shamama Nishat, Kerry Rogers, Seema Bhat, Hazem Ghoneim, Greg Behbehani, Timothy Voorhees, Karilyn Larkin, Adam Kittai, Victoria Churchill, Elizabeth George, Margaret Lamb, Dean Lee, Wing Chan, Ashley Krull, Rimas Orentas, Lynn O’Donnell, Zihai Li, Lapo Alinari, Marcos de Lima

**Affiliations:** The Ohio State University; The Ohio State University; The Ohio State University; The Ohio State University; The Ohio State University; The Ohio State University; Cleveland Clinic; The Ohio State University; Cartesian Therapeutics; Lentigen Technology Inc., A Miltenyi Biotec Company; Lentigen Technology Inc., A Miltenyi Biotec Company; Huntsman Cancer Institute; The Ohio State University; The Ohio State University; The Ohio State University; The Ohio State University; The Ohio State University; The Ohio State University; The Ohio State University; The Ohio State University; The Ohio State University; Icahn School of Medicine at Mount Sinai; The Ohio State University; Nationwide Children’s Hospital; Nationwide Children’s Hospital; Center for Childhood Cancer and Blood Diseases, Abigail Wexner Research Institute at Nationwide Children’s Hospital; The Ohio State University; The Ohio State University; Lentigen Technology Inc., A Miltenyi Biotec Company; The Ohio State University; The Ohio State University Comprehensive Cancer Center - James Cancer Hospital and Solove Research Institute; The Ohio State University; The James Comprehensive Cancer Center, The Ohio State Univ.

## Abstract

Disease recurrence is the main cause of treatment failure after CD19-directed CAR T cells and is often due to CD19 antigen loss, stability and/or coverage. To overcome single-antigen escape, we evaluated a trispecific CAR targeting CD19, CD20, and CD22 with OX40 co-stimulatory domain. Preclinical studies demonstrated potent, antigen-specific cytotoxicity in both in vitro and in vivo lymphoma models. We then conducted a first-in-human phase I trial in patients with relapsed/refractory B-cell malignancies. Fifteen patients received fresh infusions at a median vein to vein time of 7 days, at doses of 0.5–2×10^6^ cells/kg. No severe cytokine release syndrome nor immune effector cell-associated neurotoxicity syndrome occurred. Overall response rate was 50%, including complete responses in 83% of lymphoma patients. One-year overall survival rate was 61%, with durable remissions observed in lymphoma. CAR T expansion did not correlate with dose or response. T-cell exhaustion in apheresis cells correlated with progressive disease. Trispecific CAR T cells are safe and potentially active in non-Hodgkin lymphoma.

## Introduction

CD19 directed chimeric antigen receptor-modified (CAR) T cell therapy is a paradigm-changing therapeutic option in cancer immunotherapy with several FDA approved products for relapsed refractory B-cell malignancies^1–10^. Despite impressive initial response rates, disease progression remains a serious challenge. Mechanisms of treatment resistance or relapse include CAR T cell exhaustion, target antigen escape via antigen down regulation or splice mutations that prevent CAR recognition^11^. Post-CD19 CAR T relapse biopsies show that antigen loss or downregulation occurs in about 30% of patients with B-cell non-Hodgkin lymphoma (B-NHL) and 9–58% of patients with B-cell acute lymphoblastic leukemia (B-ALL)^6,7,9,12–14^. In CD19^+^ relapses, postulated mechanisms^11^ include lack of persistence, poor expansion, proliferation, and exhaustion, immunosuppressive tumor microenvironment, and loss of antigen stability and coverage.

Multi specific CAR T cells have been postulated to prevent tumor antigen escape and potentially improve treatment outcomes^15–18^. Bispecific CAR T cells targeting CD19 and CD22^19^ or CD19 and CD20^20^ have shown to be effective in inducing durable remissions. Higher response rates were observed in CD19 CAR naïve patients, highlighting the limitation of sequential targeting for B-cell malignancies.

Sequential loss of CD19 and CD22 in patients with diffuse large B-cell lymphoma (DLBCL) has also been described^21^. To further reduce the risk of antigen escape and minimize relapses, a dual CAR herein named trispecific CAR targeting CD19, CD20, and CD22 was developed. The CAR construct reported here is the first one simultaneously targeting three B-cell antigens (CD19, CD20, and CD22) requiring the presence of any one of them to trigger CAR activation and antitumor activity^22^. Additionally, this construct includes OX40 as a co-stimulatory domain^23^. OX40 receptor signaling has been shown to enhance memory T cell survival^24^ and has shown to be essential for long-term CD4+ T cell survival through Bcl-xL and Bcl-2 expression^25^. In preclinical models evaluating different combinations of co-stimulatory domains including OX40, ICOS, the most potent duoCAR structure included the OX40 co-stimulatory domain, which showed improved persistence, in vivo expansion, and proliferation of CAR Ts and demonstrated potent antitumor activity in preclinical models of B-cell malignancies with heterogeneous target antigen expression^22^.

In this first-in-human phase I trial, we evaluated the safety and preliminary efficacy of a freshly infused CD19/20/22 trispecific CAR T cell product with an OX40 co-stimulatory domain. We utilized a point-of-care manufacturing strategy with a goal vein-to-vein time of 6 days since it has been shown that shorter manufacturing times are associated with deeper responses^26^. Clinically, shorter manufacturing time prevents the need for extensive holding and bridging therapy leading to lower attrition rates in receiving CAR T as well as improved product memory/naivete leading to improved function.

## Results

### CAR construct

**Figure 1:**
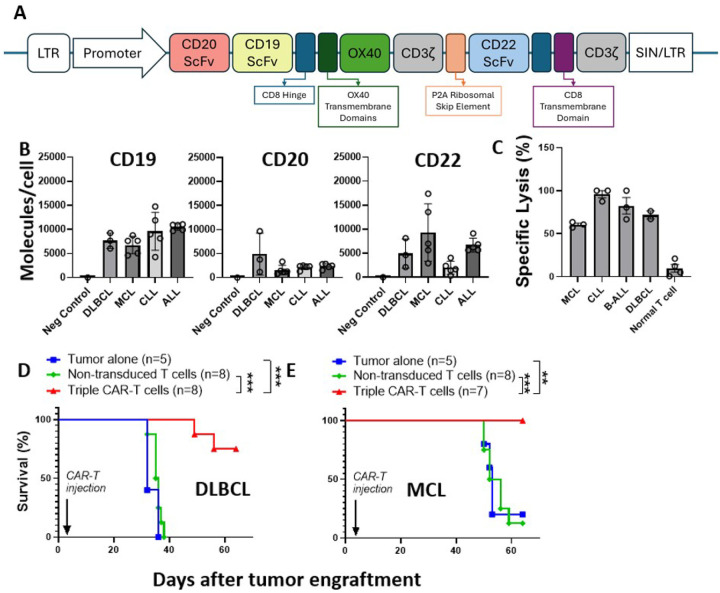
Trispecific CAR has potent in vitro and in vivo activity against B-cell lymphoma and leukemia. In-house manufactured trispecific CAR T cells demonstrate potent in vitro and in vivo effector function in leukemia and lymphoma models. **(A)** CAR Construct **(B)** Peripheral blood samples from patients with relapsed/refractory B-ALL (n=5), CLL (n=5), DLBCL (n=3), and MCL (n=5) with a white blood cell count > 50,000/μl and >80% malignant B cells were analyzed by flow cytometry. Number of molecules of target antigens was determined by the BD Quantibrite Kit. Normal T cells (n=3) were used as negative control. **(C)** Trispecific CAR T cells efficiently killed CD19/20/22 positive targets cells (MCL=3; CLL=3; B-ALL=3; DLBCL=2) but not CD19/20/22 negative normal T cells (n=4). 2.5×10^5^ trispecific CAR Ts were co-cultured with indicated target cells at an effector-to-target ratio of 5:1. After 24h, the supernatant was harvested and subjected to adenylate kinase release assay (Lonza). (D,E) Trispecific CAR T cells significantly prolonged the survival of disseminated human DLBCL **(D)** and MCL **(E)** PDXs. Tumor alone (blue, n=5), non-transduced T cells (NTT, green, n=8) were used as controls. Lymphoma cells (1×10^6^/mouse) were injected on Day 0 and trispecific CAR Ts (5×10^6^/mouse) were administered via tail vein on Day 3 post engraftment. **: p<0.01; ***p<0.001

The lentiviral vector (Lentigen Technology, Inc) was designed for simultaneous targeting of CD19, CD20, and CD22 by taking a tandem second-generation CAR construct (duoCAR20.19) comprised of scFv targeting antigens CD20 and CD19, hinge and transmembrane domain derived from CD8, OX40 co-stimulatory domain, and CD3ζ activation domain, and adding P2A ribosomal skip element and a first generation CD22-targeting CAR with anti-CD22 scFv, CD8- derived hinge and transmembrane domains and CD3ζ activation domain (duoCAR20.19.22D94)^22^. When inserted into the genome via self-inactivating, 3^rd^ generation long terminal repeats (LTR, SIN/LTR), the construct encodes two separate CAR molecules co-expressed in each transduced T cell (Fig. 1A).

### Trispecific CAR T cells demonstrate potent in vitro and in vivo activity in B-cell malignancies.

Using flow cytometry^27^, the density of target antigens on malignant B-cells from patient samples with chronic lymphocytic leukemia (CLL; n=5), B-ALL (n=5), nodal mantle cell lymphoma (MCL; n=5), and DLBCL (n=3) were measured. As shown in Fig. 1B and consistent with previously published work, CD19 is abundantly expressed in B-cell malignancies. While positive, the expression of CD20 and CD22 is more variable. In-house manufactured trispecific CAR T cells generated from healthy donor T cells (n=2) efficiently killed CD19/20/22 positive primary patient tumor samples [B-ALL (n=3), CLL (n=3), DLBCL (n=2), and MCL (n=3)] described in Fig. 1C. In addition, trispecific CAR T cells were evaluated in vivo in human DLBCL and MCL patient derived xenograft (PDX) mouse models established from two of the primary samples described in Fig. 1B. As shown in Fig. 1D,E, trispecific CAR T cells significantly prolonged the survival in each disease model. For the MCL PDX, the median overall survival (OS) was 53 days (n=5, range 50–53 days) for the tumor alone animals, 54 (n=8: range 50–65 days) for the non-transduced T cell controls (NTT), and not reached (n=8: experiment terminated at day 65) for the trispecific CAR T cell treated mice (p<0.001). For the DLBCL PDX, the median OS was 32 days (n=5, range 32–38 days) for the tumor alone animals, 35 days (n=8: range 32–37 days) for the NTT controls, and not reached (n=8: experiment terminated at day 65) for the trispecific CAR T cell treated mice (p<0.001).

### Clinical trial design

In light of the pre-clinical studies, we conducted a phase I clinical trial evaluating the feasibility of in-house manufacturing, vein-to-vein time, and safety of trispecific CAR T cells in three cohorts of patients. Cohort A included CLL, B-cell NHL with lesions < 5 cm, and B-cell prolymphocytic leukemia (B-PLL), while Cohort B included B-ALL, B-cell NHL with lesions > 5 cm, and CLL with Richter’s transformation. Cohort C included pediatric patients with any relapsed/refractory B-cell malignancy. Eligibility criteria required that patients progressed after two lines of treatment. Prior to CAR T infusion (day 0), patients received lymphodepleting chemotherapy, with cyclophosphamide 60 mg/kg/day IV given on day −6 and fludarabine 25 mg/m^2^/day IV given on days −5 through −3 for adult patients in Cohort A and Cohort B. Cohort C, which had pediatric patients, received cyclophosphamide 500 mg/m^2^/day IV given on days −6 and −5 and fludarabine 30 mg/m^2^/day IV given on days −6 through −3. Trial schema is shown in **Supplementary Fig. 1**. All patients received prophylactic tocilizumab an hour prior to CAR T infusion^28^. The primary endpoint of the study was feasibility and safety to determine maximally tolerated dose of trispecific CAR Ts. Secondary endpoints were overall response rates (ORR) including complete response (CR), partial response (PR), minimal residual disease negative remission, duration of remission, relapse rates, relapse-free survival (RFS) and one-year OS. The trial originally enrolled CAR naïve patients and later expanded to patients who previously received CD19 CAR T cells.

### Patient demographics

Six patients were treated in Cohort A (completing enrollment to dose levels 1 and 2), 9 patients in Cohort B (completing enrollment to all 3 dose levels) and 1 patient in Cohort C (dose level 2) (Table 1). Median age was 64 years (range: 11–73). Patients had received a median of three prior lines of therapy (range: 2–7). Notably, 3 patients (18.75%) had prior autologous stem cell transplant, and 2 patients (B-ALL-1, CLL with subsequent Richter’s) (12.5%) had received prior CAR T therapy. A substantial proportion presented with poor-risk features including bone marrow (68.75%) or peripheral blood disease (43.75%), and elevated lactate dehydrogenase (LDH) (87.5%). Expression of target antigens CD19, CD20, and/or CD22 was documented in 87.5%, 87.5%, and 43.75% of patients, respectively.

### Point-of-care manufacturing

Trispecific CAR T cells were manufactured at the Ohio State University (OSU) cell processing laboratory with a goal of 6-day vein-to-vein time from apheresis to infusion of fresh cells at three CAR T cell dose levels (DL): 5 ×10^5^/kg, 1 ×10^6^/kg, and 2 ×10^6^/kg. Cohort A received the entire dose as a single fresh infusion on day 0, while, Cohort B received 40% of the dose on day 0 as a fresh product and 60% on day 7 as a cryopreserved product.^29^ After safety was demonstrated in the first two dose levels, the study was amended to include a separate pediatric cohort (B-ALL and B-cell NHL) starting at dose level 2 (Cohort C) with split infusion as in Cohort B. Median transduction efficiency was 38.8% (range: 16.9%–57.6%), and median fold expansion was 5.4 (range: 1.4– 12.9). Median manufacturing time was 7 days (range: 6–10 days).

Median CD4^+^ and CD8^+^ content in non-transduced T cells (NTT) (enriched CD4^+^ and CD8^+^ T cells, prior to CAR T transduction) was 61% (range: 27 –86%) and 32% (range: 9–63%). Median CD4^+^ and CD8^+^ content in the infused CAR T product was 66% (range: 31 –89%) and 29% (range: 9–51%).

### Adverse events

As outlined in Table 2, there were no dose-limiting toxicities (DLTs). Trispecific CAR T cells were well tolerated with only mild (grade 1–2) cytokine release syndrome (CRS) occurring in 25% of patients. Among 4 patients with CRS, the median onset was 8.5 days from CAR T infusion. Of the 6 patients with B-NHL, the only patient who developed CRS (maximum Grade 2) had MCL. The other three patients who experienced CRS had B-ALL (n=2) and CLL (n=1). No patients experienced immune effector cell-associated neurotoxicity syndrome (ICANS) or Hemophagocytic lymphohistiocytosis (HLH)-like toxicities. There was no non-relapse mortality (NRM), and the overall day 100 mortality was 18.75% (due to disease progression).

### Clinical outcomes

Successful CAR T product manufacturing was achieved in 16/17 patients. Manufacturing failure occurred in one heavily pretreated patient with Richter’s syndrome despite two attempts.

The ORR including CR, PR, and minimal residual disease negative remission was 50%. The best observed response at any time was CR in 38%, PR in 13%, SD in 25%, and PD in 25%.

**Figure 2:**
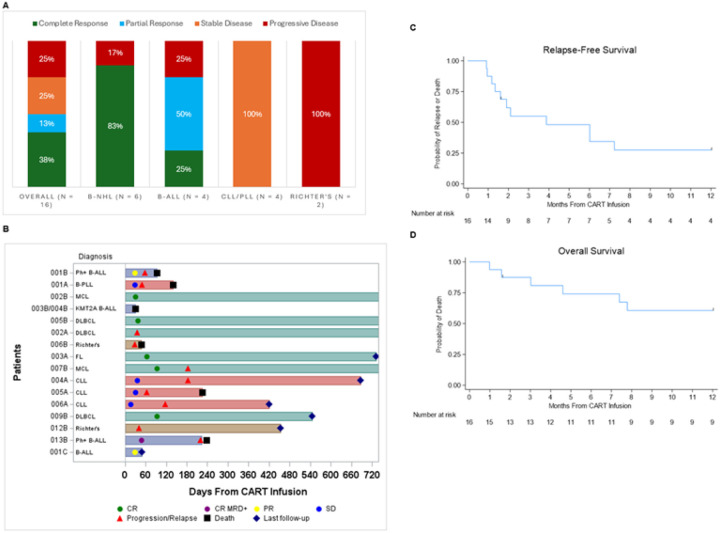
Disease response. Figure 2 shows clinical efficacy outcomes for the entire Cohort. Panel (A)disease-specific response rates. Swimmer plot (B) shows the time to response and duration of response. Panel (C) and (D) show relapse-free survival and Overall Survival from CAR T infusion, respectively, for the entire cohort.

Stratification by disease subtype revealed that CR rate was 83% in patients with B-cell NHL(N=6) and 25% in B-ALL (N=4), while all patients in the CLL/PLL group (N=4) experienced SD. No responses were seen in Richter’s transformation (Fig. 2A). Five of the six B cell NHL patients achieved CR and 4/5 remain in CR beyond 1-year post-CAR T cell infusion (Fig. 2B). Among the 6 patients with B-NHL, CR rate was 100% in MCL (n=2) and FL (n=1) and 67% in DLBCL (n=3). CR was seen across all dose levels; DL 1 (n=3), DL 2 (n=2) and DL3 (n=2). One-year relapse free survival (RFS) rate was 27.5% (95% CI 8.6%−50.7%) and one-year OS rate was 60.6% (95% CI: 32.3%−80.1%) (Fig. 2C,D). Among 6 NHL pts, one-year PFS and OS were 100% and 67% (95% CI 19%−90%), respectively. A pediatric subject with B-ALL achieved a PR and went on to receive additional infusions of trispecific CAR T cells on a single patient expanded access treatment plan. After treatment with inotuzumab, this patient achieved a CR and received an allograft. Two patients in this trial who progressed after trispecific CAR T were able to subsequently receive an allogeneic hematopoietic stem cell transplant. Responses were seen in both CD19 CAR-naïve and CD19 CAR-exposed patients. CD19, CD20, and CD22 expression pre-CAR T and, when available, at relapse is shown in **Supplementary Table 1**. CD19 expression was preserved in most patients at relapse post trispecific CAR.

### CAR T cell persistence

**Figure 3:**
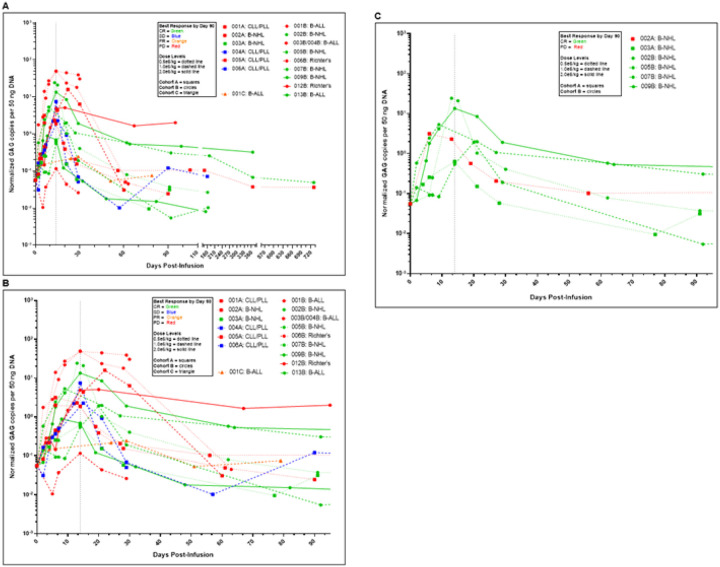
Trispecific CAR T Cell Persistence by GAG gene PCR CAR T cell persistence by GAG gene qPCR shown by days post-infusion with all data points **(A)** and by days post-infusion up to 90 days **(B)**. The data is grouped by best response by day 90, with dose levels and cohorts indicated by line type and icon. There was no statistically significant difference in normalized GAG copies per ng DNA between responders and non-responders on post-treatment days 14, 30, or 90. Panel **(C)** B-NHL patients CAR T cell persistence by qPCR shown by days post-infusion up to 90 days. The data is grouped by best response by day 90, with dose levels and cohorts indicated by line type and icon. Responders (green) and non-responder (red) are shown.

Trispecific CAR T cells were detected in the peripheral blood by quantitative GAG gene PCR. There were no statistically significant differences in levels as measured by normalized GAG copies per ng DNA between responders and non-responders on post-treatment days 14, 30, or 90 (Fig. 3A–C). Peak expansion of CAR T cells occurred on days 14–15 for all but three patients who peaked by day 20–29. Peak normalized GAG copies per 50 ng DNA ranged from 0.12 to 50.03 (median 2.28; mean 11.06 ± 15.92 standard deviation). There were no significant differences across different B-cell malignancy subtypes and among the dose levels or responders vs non-responders. Similar results were obtained when expansion was measured by area under the curve (AUC) up to day 30 for 16 patients and up to day 90 for 11 patients (**Supplementary Fig. 2**).

### Non-transduced T (NTT) cell phenotype correlates with clinical responses.

**Figure 4:**
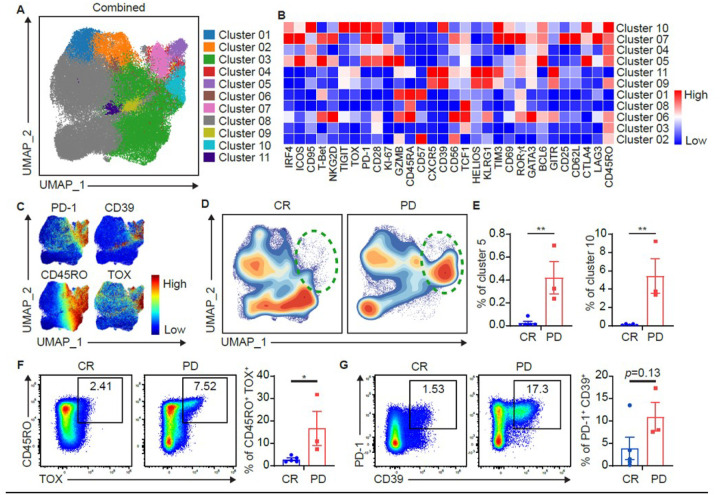
Immunophenotypic and functional characteristics of non-transduced CD8+ T cells in responders and non-responders in lymphoma patients. Non-transduced T cells (NTTs) used to manufacture trispecific CAR T were analyzed via spectral flow cytometry. **(A)** UMAP dot plot of CAR^**+**^ CD8^**+**^ T cells from the total cohort (n=8). **(B)** Clustered heatmap showing key marker expressions on differentially abundant CAR^**+**^ CD8^**+**^ clusters. Color scale was determined by median normalization of each individual marker with blue representing low expression, white representing median expression and red representing high expression. **(C)** Expression plots of phenotypical markers present on CAR^**+**^ CD8^**+**^ T cells. **(D)** Contour UMAP plots of CAR^**+**^ CD8^**+**^ T cells in CR vs. PD cohorts. **(E)** Box plots showing clusters percentages of CAR^**+**^ CD8^**+**^ Cells in CR vs. PD cohorts. Left: Cluster 5. Right: Cluster 10. For **(F)** and **(G)**, 2D flow plot analysis was performed on spectral flow cytometry data from all patient NTT samples. **(F)** Representative 2D flow cytometry plots showing individual patient CD45RO and TOX expression on NTTs and corresponding box plot quantification in CR vs. PD cohorts. **(G)** Representative 2D flow cytometry plots showing individual patient PD-1 and CD39 expression on NTTs and corresponding box plot quantification in CR vs. PD cohorts. Box plots in **(E)**, **(F)**, and **(G)** show quartiles with bands at the median; whiskers indicate 1.5 interquartile range; all observations overlaid as dots. p values are from two sample t test. p < 0.05 = *. p < 0.01 = **.

High-dimensional spectral flow cytometry profiling was performed on lymphoma patients on NTT, infusion products, and on day 7 and 14 samples. B-cell NHL patients were selected for analysis to minimize variances in response criteria and disease heterogeneity. We first analyzed CD8^+^ NTT cells: UMAP-based clustering identified 11 distinct clusters of CD8^+^ T cells. Notably, Cluster 10 was enriched in patients that did not respond to therapy (PD) (Fig. 4A,B) and was characterized by increased expression of exhaustion markers such as PD-1, TIM-3, CD39, TOX, TIGIT, and CTLA-4 (Fig. 4C). In contrast, Cluster 3 was enriched in patients who achieved a complete response (CR) and displayed a less activated phenotype (TCF1^+^)^30^ (Fig. 4D). These findings were confirmed in a separate analysis via 2D plotting which also revealed enrichment of CD8^+^ T cells co-expressing CD45RO and TOX, PD-1, and CD39 in PD patients (Fig. 4 F, G).

A similar analysis identified 12 distinct clusters of CD4^+^ T cells (**Supplementary Fig. 3**). Cluster 5 was found to be more abundant amongst PD patients (**Supplementary Fig. 3E**) and was characterized by expression of GATA3 as well as increased exhaustion markers (i.e. PD-1, CD39, TIGIT, and TOX). Although not statistically significant, CD4^+^ T cell clusters with increased numerical frequency in the CR cohort were characterized by a less activated phenotype as well (TCF1^+^).

Together, these results suggest that pre-existing differences in T cell exhaustion phenotypes in enriched CD4/8 ^+^ T cells used to manufacture the trispecific CAR T cell product may be predictive of post-trispecific CAR T cell therapy outcomes in patients with B-cell NHL.

### CAR^+^ T cell phenotype in infused products did not correlate with clinical responses.

**Figure 5:**
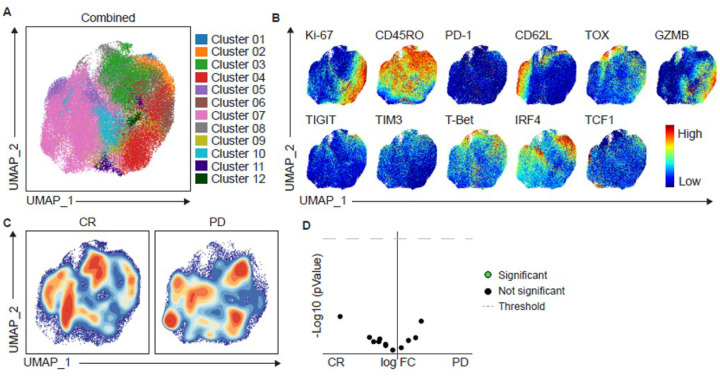
Immunophenotypic and functional characteristics of CD8+ CAR+ T cells in Trispecific CAR T infusion products. Patient’s trispecific CAR T infusion products were analyzed by spectral flow cytometry. **(A)** UMAP dot plot of CAR^**+**^ CD8^**+**^ T cells from the total cohort (n=8). **(B)** Expression plots of phenotypical and functional markers present on CAR^**+**^ CD8^**+**^ T cells. **(C)** Contour UMAP plots of CAR^**+**^ CD8^**+**^ T cells in CR vs. PD cohorts. **(D)** Volcano plot plots of all clusters differentially expressed in responders (CR) vs. non-responders (PD) lymphoma patients. P ≤0.05 was considered significant.

CD8^+^ CAR^+^ T cells within the infusion product from patients with B-cell NHL were profiled using singlecell clustering and UMAP visualization. Although 12 phenotypically distinct clusters were identified (Fig. 5A), no statistically significant differences in CAR^+^ T cell composition were observed between responders vs non-responders (Fig. 5B–D). Similar analysis of CD4^+^ CAR^+^ T cells within the infusion product from patients with NHL was performed and revealed no differences in CAR^+^ T cell phenotype (**Supplementary Fig. 4**). Therefore, the phenotypic profile of CAR^+^ T cells at the point of infusion did not correlate with clinical response, in contrast to the differences observed in the source NTT material.

### Terminally exhausted CD8^+^ CAR T cells are increased in non-responders at Day +7 post-CAR T cell infusion.

Single-cell clustering revealed 12 distinct CD8^+^ CAR^+^ T cell populations (**Supplementary Fig. 5A,B**). Visualization of cells through contour plots revealed areas of the UMAP in which CD8^+^ CAR^+^ T cell events were more prevalent amongst responders (**Supplementary Fig. 5C**). Cluster 3 was significantly increased in responders while Clusters 1, 9, and 11 were significantly increased in non-responders (**Supplementary Fig. 5D**).

The phenotypic signature of the non-responders-associated clusters included high expression of exhaustion-related markers such as PD-1, TOX, IRF4, TIGIT, and CD39, along with elevated Ki67 and high levels of GZMB. These features suggest a population of CD8^+^ CAR^+^ T cells that is highly activated as well as populations which are terminally differentiated. In contrast, Cluster 3, which was more prevalent in responders, consisted of less activated CD8^+^ T cells characterized by low PD-1 expression, intermediate GZMB, and low Ki67.

This was further analyzed via two sample tests (box plots analysis) which found Cluster 3 was significantly increased in responders (**Supplementary Fig. 5E**). Although not statistically significant, Cluster 11 was found to be increased in the non-responder subgroup (**Supplementary Fig. 5E**).

Visualization of CD4^+^ CAR^+^ T cells at Day +7 post-CAR T infusion through contour plots revealed areas of the UMAP in which CD4^+^ CAR^+^ T cell events were more prevalent in CR vs. PD patients (**Supplementary Fig. 6**). Volcano plot analysis revealed Clusters 1, 2 and 6 were more abundant amongst non-responders (**Supplementary Fig. 6D**). These clusters were characterized by higher levels of proliferation (Ki67) and exhaustion related markers including PD-1 concurrently with TOX, CD39 and TIGIT. A population of Tbet^+^ CD4^+^ CAR^+^ T cells was also present, indicating a higher level of Th1-like subtype of CAR T cells at this timepoint. There were no clusters significantly increased in the CR cohort.

Together, these results suggest that increased activation and proliferation, as well as increased markers of exhaustion at Day 7 on CAR^+^ T cells was associated with NHL progression with this CAR T cell product.

### Day 14 CD8^+^ CAR^+^ T cell phenotypes and disease response

Single cell clustering analysis was used to analyze CD8^+^ CAR^+^ T cells from patients with B-cell NHL and resulted in 12 clusters (**Supplementary Fig. 7A,B**). Visualization of cells through contour plots revealed areas of the UMAP in which CD8^+^ CAR^+^ T cell events were more prevalent amongst responders (**Supplementary Fig. 7C**). Cluster 9, 10, and 12 were significantly increased in the CR cohort while Cluster 1 was increased in the PD cohort (**Supplementary Fig. 7D**). This was further analyzed via box plots analysis which confirmed Cluster 10 to be significantly increased in the CR cohort (**Supplementary Fig. 7E**).

The CR-associated clusters displayed characteristics of less activated T cells lacking PD-1, and moderate GZMB levels, as well as T cells with a memory-like phenotype that may support longer-term persistence^31–33^. Interestingly, Ki67 expression in these cells was higher at Day 14 samples in responders compared to Day 7, suggesting ongoing proliferation in responding patients. Consistent with Day 7 data, the PD-associated Cluster 1 displayed sustained high expression of exhaustion markers (PD-1, TOX, TIGIT, IRF4) and lower Ki67 expression compared to day 7, indicating an earlier acquisition of exhaustion state in non-responders.

On Day 14, similar to observations in CD8^+^ CAR T cells, CD4^+^ CAR^+^ Cluster 2 (high expression of exhaustion markers) was significantly enriched in non-responders (**Supplementary Fig. 8**).

## Discussion

Here we describe the results of a first-in-human clinical trial with a trispecific CAR T cell product targeting CD19, CD20, and CD22 and an OX40 co-stimulatory domain. It has been suggested that fresh cell infusions are preferable^34,35^, and accordingly we demonstrate the feasibility of rapid manufacturing time and infusion of a fresh product in patients with B-cell malignancies.

Safety profile was favorable with only low-grade CRS and no instances of HLH or ICANS. In a study of 38 patients treated with bispecific CAR T cells targeting CD19 and CD22, ICANS occurred in 37%. These bispecific CAR T cells also demonstrated reduced intracellular cytokine production when stimulated with CD22 versus CD19^19^. We speculate that the lack of HLH observed in our study might be due to the suboptimal expansion of trispecific CAR T cells and the lack of a costimulatory molecule associated with the anti-CD22 CAR T component. Contrary to prior studies showing correlation with early peak expansion of CAR T cells and subsequent responses^35^, there was no correlation between day 14 CAR T cell expansion and response. Furthermore, there was no correlation between dose levels and day 14 peak expansion. Despite suboptimal expansion of trispecific CAR T cells compared to CD19 CAR T cells and bispecific CAR T cell products^19,36,37^, there was significant activity in B-NHL with 100% CR rate in MCL and FL and 67% CR rate in DLBCL, notwithstanding the small number of patients. In comparison, CR rates after axi-cel, tisa-cel, and liso-cel in relapsed/refractory B-cell lymphomas were 55% (95% CI, 43–68), 32% (95% CI, 17–51), and 53% (95% CI, 23–59), respectively^38^. Commercial CD19 targeted CAR results in ORR rates of 53% and CR rates of 39% in third line settings vs 87% and 74% respectively when administered in second line setting for B-NHL^39^. In our study, durable responses were seen only in B-NHL, suggesting a strong efficacy signal for this population. Trispecific CAR T cells had only modest activity against B-ALL, CLL and CLL in Richter’s transformation.

OX40 co-stimulation has been reported to lead to enhanced CAR T cell proliferation, improved tumor killing, and longer persistence^22–25^. Interestingly, OX40 binds to heparan sulfate which is highly expressed on the surface of malignant cells. This interaction has been shown to enhance CAR T cell tumor infiltration in preclinical models of solid tumors^40^. In addition, OX40 is involved in T cell homing^41^ suggesting that efficient CAR T cell homing to lymph nodes may result in low CAR T cell counts in the peripheral blood as previously shown^42^. While the explanation for lack of responses in CLL and suboptimal response in B-ALL is certainly multifactorial, we hypothesize that the presence of an OX40 co-stimulatory domain may play a role in the differential response in leukemia vs lymphoma.

Interestingly, non-transduced T cells from non-responder lymphoma patients were enriched for what appears to be tumor-reactive CD8 T cell populations, aligned with an intermediate/cytolytic subset of exhausted T cells^43–45^. The presence of these potentially tumor-reactive TCR clones in NTT (and early post-infusion samples) may interfere with CAR T cell activity, potentially contributing to disease progression through disruption of CAR T cell signaling, accelerating terminal differentiation and exhaustion of CAR T cells. Future studies with single cell TCR-sequencing analysis to explore clonal overlap and antigen specificity will provide further characterization of these clones.

Together, these results suggest that pre-existing differences in T cell phenotypes in a subset of T cells used to manufacture the trispecific CAR T cell product may be predictive of therapy outcomes in patients with lymphoma. Trispecific CAR T cells from patients that did not respond had an exhausted phenotype. It is unclear if this is due to antigen overstimulation given that three antigens are targeted simultaneously or to the OX40 co-stimulatory domain is unknown.

Unlike other studies^46^, we did not find a correlation between CAR^+^ T cell phenotype in infused products and clinical responses in lymphoma patients^42,47,48^. After treatment, early proliferation of CD8^+^ CAR T cells led to early expression of exhaustion markers and poorer response rates. Additionally, clusters of PD1^+^ cells with a memory phenotype and with moderate levels of GZMB were associated with remission and may denote longer term persistence of functionality of CAR T cells. In general, marker characterization on CAR^+^ CD8^+^ T cells thus may act as a potential biomarker of efficacy with this CAR T product.

The role of CD4^+^ CAR T cells has not been described well in CAR T cells, outside their role as potential suppressors of CAR T function as CAR^+^ Tregs^42,48^. However, it is known that from a physiologic standpoint, there exist cytotoxic CD4^+^ T cells that can kill tumor and prior evidence has shown that CD4^+^ support of CD8^+^ T cells may be crucial to efficient killing^49^. Here, the changes that occurred in CD4^+^ cells both prior to manufacturing and after infusion did not reach statistical significance.

Relapse patterns associated with monospecific CARs targeting CD19^1–3^ or CD22^14,50^ frequently show target antigen downregulation^16,51–54^. Relapses in this study were predominantly CD19^+^. This finding along with low expansion of CAR T cells and exhausted phenotype in non-responders suggest that lack of persistence and intrinsic defects in CAR T cells were most likely the cause of relapse rather than antigen pressure or downregulation.

This phase I study enrolled a heterogenous population of patients with hematologic malignancies. Given efficacy signal in B-cell NHL with minimal toxicity, exploration of trispecific CAR T in this disease population appears justifiable.

## Methods

### Preclinical Studies:

#### Primary lymphoma and leukemia patient samples

Cryopreserved primary lymphoma and leukemia patient samples were obtained from the Ohio State University (OSU) Hematology Tissue Bank Shared Resource following written, informed consent in accordance with the Declaration of Helsinki under a protocol approved by the OSU IRB. Cryopreserved peripheral blood samples obtained from patients with B-ALL, CLL, nodal MCL and DLBCL in leukemic phase were cultured in standard RPMI1640 with 10% FBS and 5% CO2 and used immediately upon thawing. Cryopreserved normal human T cells isolated from peripheral blood mononuclear cells (PBMCs) using EasySep Human T Cell Isolation Kit (STEMCELL Technologies, Vancouver, Canada) were used as negative control.

#### Flow cytometry

Cryopreserved de-identified lymphoma and leukemia patient samples were thawed and washed with PBS. A cell count for total cell number and viability was done using hemocytometer. 1 million cells were taken for quantitative flow for each patient sample. Samples were stained for human CD19PE, clone HIB19 (catalogue# 555413 BD), human CD20 PE clone H1 (catalogue#561174 BD), human CD22 PE clone HIB22 (catalogue# 562859BD); mouse IgG1K (catalogue# 555749 BD) was used as an isotype control; live/dead fixable Near-IR from Thermo Fisher (catalogue# L34981) was used to distinguish between live and dead cells. Quantitative flow cytometry was done using BD Quantibrite^™^Beads, PE Fluorescence Quantitation Kit purchased from BD Bioscience. Briefly, BD Quantibrite PE Tube was reconstituted in 0.5mL FACS buffer. Beads were run on BD LSRFortessa Flow Cytometer, setting the threshold for FSC or SSC, and 10,000 events were collected. The gate was adjusted around bead singles on FSC-H vs SSC-H plot. Singlet bead population was analyzed using histogram plot of FL2-H. After instrument adjustment, patient samples were acquired. Manual analysis was done using the geometric means of each histogram for the four bead peaks and Linear regression of Log_10_ per bead against Log_10_ fluorescence was plotted. Calibrated beads contain a specific number of fluorophore molecules bound per bead and are used to standardize and convert the mean fluorescence intensity (MFI) in flow cytometry into a count of fluorophores. These allow for the calculation of the number of target antigens per cell when using antibodies under saturating conditions, considering the Fluorophore to Protein Ratio (F:P) of each antibody. A calibration curve correlating instrument detection channel values and standardized fluorescence intensity units on constructed with R2 of 0.9995. With the F:P ratio of 1 for CD19/CD20/CD22 antibodies, the correlation equation was used to calculate the target antigen density on tumor cells from the MFI obtained on the same day with settings according to the manufacturer’s instruction.

#### Cytotoxicity assay

The cytotoxicity of the CAR T cells was performed with ToxiLight^™^ non-destructive cytotoxicity bioassay kit (Lonza) and as manufacturer’s instruction described^55^. Briefly, primary lymphoma and leukemia patient samples (lymphoma % ranged from 73.7% to 98.7%) were co-cultured with trispecific CAR T cells or NTT cells at E:T ratio of 5:1 for 24 h. Lysis Buffer was then added to the culture at a 1:2 ratio of lysis reagent to sample volume and incubated for 10 min at room temperature. The volume in the control wells was adjusted with the provided Tris AC buffer. The cell supernatant from each well was harvested and incubated with the provided substrate for 5 min before the plates were read for bioluminescence with a Synergy HT microplate reader (Biotek, VT).

#### In vivo studies

All animal studies were approved by the OSU Institutional Animal Care and Use Committee. Mice were purchased from the Jackson Laboratory (Bar Harbor, ME). Four to five mice were housed per cage. Nutritional supplementation was provided to all animals, including with DietGel Boost (ClearH20, 72-04-5022, Westbrook, ME) and hydration with Hydrogel (ClearH20, 70-01-1082). Throughout all animal studies, monitoring was performed daily by both the researchers and institutional employees. Mice were euthanized upon meeting specific early removal criteria (ERC), including systemic signs of tumor burden (>20% weight loss in < 1 week, hind limb paralysis, respiratory distress, weight loss, ruffled coat, or distended abdomen).

The MCL and DLBCL patient derived xenograft (PDXs) were developed in Dr. Alinari’s lab by intravenous tail vein engraftment of unselected PBMCs from 2 lymphoma patients (1 MCL and 1 DLBCL) into 5–7-week-old female NSG mice (NOD.Cg-Prkdcscid Il2rgtm1Wjl/SzJ, Jackson Laboratory). Splenic lymphoid/MCL cells harvested from tumor-bearing mice were serially re-engrafted, and lymphoma cells isolated from passage 3 animals were used in the present study. Four-week-old female NSG mice were engrafted with 1 million MCL and DLBCL PDX cells suspended in PBS via intravenous tail vein injection. In-house manufactured trispecific CAR T cells (5×10^6^/mouse) were administered intravenously on Day 3 post-engraftment. Additional animal groups included tumor alone as well as NTT-treated animals.

Mice were visually monitored daily and weighed twice weekly. Disease burden was assessed periodically via evaluation of human CD19^+^ (and CD5^+^ for MCL) lymphocytes in the peripheral blood by flow cytometry. Blood from nongrafted NSG mice was used as a negative control. For all mice, survival was considered the primary endpoint, and euthanasia was performed upon development of ERC.

### Clinical Studies:

#### Trispecific product manufacturing

Patients underwent apheresis and lymphodepletion began the day after apheresis. Trispecific CAR T cell manufacturing was initiated the day after apheresis and utilized the duoCAR20.19.22D94 lentiviral vector and the CliniMACS Prodigy device (Miltenyi Biotec). The apheresis product was assessed for microbial contamination and enumerated using a hematology analyzer, and CD4^+^ and CD8^+^ percents are determined using flow cytometry in a standard immunophenotyping panel.

The Miltenyi Biotec CliniMACS Prodigy System uses the TS520 tubing set for closed system manufacture of CAR T cells including selection, activation, transduction, and expansion. CD4^+^ and CD8^+^ T cells were enriched from the apheresis product using GMP-grade CliniMACS CD4 and CD8 reagents and analyzed by flow cytometry. These enriched CD4^+^ and CD8^+^ T cells are referred to as non-transduced T cells (NTTs). NTTs were activated using MACS GMP T Cell TransAct for 18–30 hours and then transduced with the duoCAR20.19.22D94 lentiviral vector for 24 hours. Subsequent dilution and media exchange steps were fully automated through the Prodigy program.

Expansion of activated and transduced T cells to meet required clinical dose occurred through harvest in serum-free and xeno-free TexMACS GMP Medium supplemented with IL-7 and IL-15 (both at 12.5 ng/mL). Beginning 5 days after culture initiation, a viable cell count and flow cytometry testing for CAR^+^ T cells were performed using sterile accessible ports in the Prodigy to determine if protocol-specified CAR T cell dose had been achieved. If the patient’s assigned dose had not been met, sampling and testing was repeated daily until sufficient cells were available for harvest. Once the patient’s CAR T cell dose was reached, culture samples were collected to test for mycoplasma, replication-competent lentivirus (RCL), and vector copy number (VCN), PCR testing was initiated, and harvest planned the following morning. For the final harvest, CAR T cells were formulated in Plasma-Lyte with 5% Human Serum Albumin (HSA) at a density of up to 1 × 10^8^ total nucleated cells/mL. Samples of the final harvested product were obtained to evaluate cell content, viability, immunophenotype, percent CAR T cells, gram stain, endotoxin, and microbial culture. After CAR^+^ cell yield was calculated, the final product was divided into fractions for fresh infusion and/or cryopreservation based upon the patient’s study cohort assignment, including cryovials frozen for later correlative analysis. Once harvested and all release testing was completed and reviewed, the CAR T cell product was released and distributed to the patient bedside for fresh infusion. For patients in cohort B and C, 60% of the cell dose was frozen and infused on day +7.

#### Flow cytometry (Clinical Product/Release Testing)

Final product release testing for determination of the transduction efficiency of the trispecific CAR T cells was determined by flow cytometry using a two-step staining process. The cells were incubated for 1 hour at 1-10°C with His-tagged CD22 protein (Acrobiosystems, catalog # CD2-H52H8) and biotin-tagged CD19 CAR detection reagent (Miltenyi Biotec, catalog # 130-129-550). Following incubation, cells were washed and stained for 15 minutes at 1-10°C with fluorescently labeled secondary antibodies (His Antibody, PE, Miltenyi Biotec, catalog # 130-120-718 and Biotin Antibody, APC, Miltenyi Biotec, catalog # 130-110-952) along with immunophenotyping antibodies (Miltenyi Biotec, REAfinity^™^) to CD45 (clone REA747/5B1, catalog # 130-110-637), CD3 (clone REA613/SK7, catalog # 130-113-138), CD4 (clone REA623/SK3, catalog # 130-113-230), CD8 (clone REA34/HIT8a, catalog # 130-110-680) and the viability dye 7-AAD (BD Biosciences, catalog # 559925). Controls included a fluorescence minus two (FMT; lacking CD22 protein, His tag and CAR19 detection reagent) along with fluorescence minus one (FMO) controls for each CAR detection reagent, creating a four tube panel for CAR detection. After washing, the samples were reconstituted and acquired on the Beckman Coulter CytoFLEX flow cytometer and data analyzed with Beckman Coulter Kaluza Analysis Software. A sequential gating strategy (singlets, debris exclusion, CD45^+^ then viability) is utilized prior to determining the %CD3 and % CAR. The FMT control, FMO controls, and positive population clusters were used to determine the transduction efficiency of the product.

#### Clinical study design

Patients were enrolled to a phase I dose-finding study (NCT05418088). The clinical protocol is available in the Supplementary material. The primary endpoint of the study was feasibility and safety to determine maximally tolerated dose of trispecific CAR T cells. Secondary endpoints were ORR including CR, PR, minimal residual disease negative remission, duration of remission, relapse rates, PFS and one year OS. Response was assessed at 30 days, 3, 6 and 12 months and were assessed by Lugano criteria for NHL, IWG criteria for CLL and B-PLL as listed in the protocol (Supplementary material).

#### Correlative studies

Cryopreserved NTTs and trispecific CAR T infusion product samples cryopreserved at the time of harvest were utilized for correlative studies. In addition, cryopreserved PBMCs derived from Ficoll-Paque separation of blood samples drawn at timepoints pre- and post-CAR T infusion (Day −6, Day 0, Day +2, 4, 6, 14, 21, 30, 90, 6 months and at 12 months) were used for correlative analysis.

#### Quantitative PCR for in vivo CAR T detection

Genomic DNA was extracted from thawed peripheral blood mononuclear cells using the DNeasy Blood and Tissue kit (QIAGEN). Quantitative PCR (qPCR) was performed using the MACS^®^ COPYcheck kit (Miltenyi Biotec) on the ABI 7500 Fast Real-Time PCR system (Thermo Fisher Scientific). Primers amplify a modified *psi-gag* sequence present in the integrated provirus and the human reference gene *PTBP2* for normalization, with detection via hydrolysis probes. The *gag* gene used as a marker for integrated CAR transgene is directly downstream from a psi (ψ) packaging signal and is a synthetically modified, partial *gag* sequence. Each PCR reaction contained Taqman Fast Advanced Master Mix, 0.40 μM of primers and probes, and 250 ng patient DNA, except samples with low circulating cell counts which contained 68–238 ng. For persistence of CAR T cells post-infusion, the *gag* and PTBP2 copies/μL results were multiplied by the test sample’s DNA concentration (ng/μL) to determine copies per ng of the transgene and the reference gene. *Gag* copies/ng was then divided by *PTBP2* copies/ng to calculate the normalized *gag* copies per ng DNA and per 50 ng DNA.

#### Spectral flow cytometry

A 40-color CAR T cell spectral flow cytometry panel was developed to identify features of the immune cells that may correlate with clinical outcomes. The panel (**Supplementary Table 2**) contains markers used to identify CAR T cell phenotype and function and characterize naivete, memory, activation, exhaustion, and senescence. A G4S linker (E7O2V; Cell Signal) detection reagent specific to the G4S linker region between single chain variable fragments present on the tri-specific CAR T construct was used to identify CAR^+^ T cells. Cells were analyzed by spectral flow cytometry as previously described^47^.

NTTs derived from apheresis products, trispecific CAR T infusion product, and PBMCs at Days 7, 14, and 30 post-CAR T infusion along with healthy donor PBMCs were analyzed.

To minimize variances in response criteria and disease heterogeneity, only the eight patients diagnosed with lymphoma were studied. Spectral flow cytometry results were correlated with achievement of CR by 6 months which has been shown to associate with long term outcomes^2,42,48,56^. The gating strategy used to identify CAR^+^ CD8^+^ or CAR^+^ CD4^+^ T cells is shown in **Supplementary Fig. 9**.

#### High-dimensional spectral flow cytometry data analysis

Flow cytometry data was uploaded to web-based software OMIQ for analysis (https://app.omiq.ai/) and analyzed as previously described^47^. For further validation, flow cytometry data were reevaluated using FlowJo (BD) by creating 2-dimensional (2D) plots and using concomitant statistical approaches.

### Statistical Analyses:

For preclinical in vitro experiments, two-sample t-test or ANOVA was used for two group comparisons or for multiple group comparisons, respectively. For paired or correlated data, such as cells from the same subjects treated with different treatment conditions, paired t-tests or linear mixed effects models were used to adjust the correlation among observations from the same object. For preclinical in vivo studies, log rank test was used for comparison and survival curves were estimated with Kaplan–Meier survival curves. P-values were adjusted for multiple comparisons by Holm’s procedure when needed. A p-value less than 0.05 for two group comparisons or after adjustment for multiple comparisons was considered statistically significant.

Patient clinical characteristics were summarized using medians and ranges for continuous variables and frequencies and percentages for categorical variables. Response rates were calculated as proportions of achieving the response among treated, and the bar graph was generated in Excel. Response duration was calculated from the date of best response to the date of death or relapse, censoring those without a relapse or death at the last follow-up. PFS was calculated from CAR T infusion to the date of death or relapse, censoring those without a relapse or death at the last follow-up. OS was calculated from CAR T infusion to death, censoring those alive at the last follow-up. Stata 18 was used for the analysis. The time to clinical response was displayed in a swimmer’s plot generated using SAS sgplot.

For qPCR data measuring CAR T cells, normalized GAG copies per ng DNA were log normalized. Linear mixed-effects models were used to model the log normalized data between CR vs progressive disease (PD) at three time points with patients being the random effect. In addition, the area under the curve (AUC) of the log normalized data between day 0 and day 90 was compared between CR and non-CR by two-sample t-test. Holm’s procedure was used to adjust p-values of multiple comparisons.

## Supplementary Files

This is a list of supplementary files associated with this preprint. Click to download.

- SupplementaryFiguresandTables09JAN2026.docx
- OSU21170Protocolv1131OCT25appr05JAN26.pdf
- RSVasu.pdf

## Figures and Tables

**Table 1:** Patient Characteristics (n=16)

Age, median (range)	64	11–73
	N	%
Diagnosis		
B-ALL	1	6.25
KMT2A B-ALL	1	6.25
Ph+ B-ALL	2	12.5
B-PLL	1	6.25
CLL	3	18.75
DLBCL	3	18.75
FL	1	6.25
MCL	2	12.5
Richter’s	2	12.5
Diagnosis, re-grouped		
B-ALL	4	25
CLL/PLL	4	25
B-NHL	6	37.5
Richter’s	2	12.5
Prior lines of therapy, median (range)	3	2–7
Prior Autologous-SCT	3	18.75
Prior Allogeneic -SCT	1	6.25
Prior CAR T	2	12.5
Bulky disease (>=5cm)	7	43.75
Bone marrow disease	11	68.75
Peripheral blood disease	7	43.75
Elevated LDH	14	87.5
Elevated Ferritin	7	43.75
CD19+	14	87.5
CD20+	14	87.5
CD22+	7	43.75
1 of CD19+, CD20+, CD22+	2	12.5
2 of CD19+, CD20+, CD22+	9	56.25
3 of CD19+, CD20+, CD22+	5	31.25

**Abbreviations**: SCT: stem cell transplant; ALL: acute lymphocytic

Leukemia; PLL: prolymphocytic leukemia; CLL: chronic lymphocytic

Leukemia; NHL: non-Hodgkin’s lymphoma; MCL: mantle cell lymphoma;

FL: follicular lymphoma

**Table 2:** Toxicity and other clinical outcomes.

	N	%
CRS grade		
0	12	75
1	1	6.25
2	3	18.75
ICANS grade		
0	16	100
HLH		
0	16	100
ANC>500 by Day 35	12	75
Time to ANC>500, Median (Range)	13	9–32
ANC>1000 by Day 60	12	75
Time to ANC>1000, Median (Range)	15.5	9–53
Platelets < 20K by Day 30	3	18.75
n/a	10	
Platelets < 50K by Day 30	5	31.25
n/a	5	
IgG > 400 mg/dL	2	13.3
Infection by Day 100	2	12.5
Infection grade 3+	2	12.5
Other malignancy (none associated with CAR-T)	3	18.75
Death by Day 30	1	6.25
Death by Day 100 (all due to disease progression)	3	18.75
Non-relapse mortality	0	0

**Abbreviations:** CRS: cytokine release syndrome; ICANS: immune effector associated neurotoxicity syndrome; ANC: absolute neutrophil count; IgG: immunoglobulin; HLH: hemophagocytic lymphohistiocytosis
